# Collection of vaccination data in the German National Cohort: Findings of a feasibility study in three study centers

**DOI:** 10.1007/s00103-014-2050-0

**Published:** 2014-10-08

**Authors:** A. Schultze, M.K. Akmatov, S. Castell, A. Karch, W. Ahrens, K. Günther, H. Schlenz, D. Flesch-Janys, N. Obi, F. Pessler, G. Krause

**Affiliations:** 1 Department for Epidemiology, Helmholtz Centre for Infection Research, Inhoffenstraße 7, 38124 Braunschweig, Germany; 2 Leibniz Institute for Prevention Research and Epidemiology – BIPS, Bremen, Germany; 3 Universitäres Cancer Center Hamburg, Universitätsklinikum Hamburg-Eppendorf, Hamburg, Germany; 4 TWINCORE Center for Experimental and Clinical Infection Research, Hannover, Germany; 5 Hanover Medical School, Hannover, Germany

**Keywords:** Vaccination certificate, Vaccination data collection, Population-based study, Vaccination status, Impfpass, Impfdatenerhebung, Bevölkerungsbasierte Studie, Impfstatus

## Abstract

**Background:**

Data about the vaccination status of participants are required in epidemiological cohort studies whenever infection or immunity is considered as potential exposure or outcome. Within Pretest 2 of the German National Cohort (GNC) we therefore investigated the acceptance and feasibility of extracting vaccination status from vaccination certificates provided by the participants of the study.

**Methods:**

This study was conducted in three study centers (Bremen, n = 73; Hamburg, n = 200; Hannover, n = 193). In order to test if an additional reminder would prevent participants from forgetting their vaccination certificates at home persons willing to participate in Pretest 2 were randomly assigned to one of three invitation groups (IG). About one third of the participants received either no further reminder (IG1), a reminder card together with the appointment letter (IG2) or a separate reminder card 4 days before the appointment (IG3). At the study center, vaccination data were scanned or copied and entered into a database using a unique identification number. Participants were also asked to fill in a short questionnaire to assess the completeness of the provided vaccination data. Additionally, in one of the three participating study centers, general practitioners (GP) were asked to provide vaccination data from their records following respective participants’ consent. Finally, we compared the influenza data from the vaccination certificates with the influenza data obtained from participants in Pretest 2 by use of a self-administered questionnaire (ID-Screen).

**Results:**

Due to different starting dates of the study the intended reminder procedure was implemented only in Hamburg and Hannover. In Hamburg, significantly more vaccination certificates were submitted by the group which received the reminder card separately 4 days before the examination (IG3) compared to IG1 and IG2 (*p* = 0.04). In Hannover, in contrast, most vaccination certificates were brought by those who received the reminder card together with the appointment letter. Overall, the use of a reminder card had a positive but not significant effect as 89 % (185/209) of participants who received the reminder card submitted vaccination data versus 81 % (84/104) of participants who did not receive any reminder card (*p* = 0.06). Of all Pretest 2 participants in Hannover, 62 % (120/193) gave written consent for data collection by the GPs. In total, 114 practices were contacted of which 49 (43 %) sent vaccination data. All in all, 360 vaccination certificates with 5065 documented vaccinations were entered into a database, of which 4830 (95 %) were valid for analysis covering a period from 1946 to 2012. The comparison of influenza vaccination data from vaccination certificates to the remembered data from a self-completed questionnaire showed an agreement of data in 46 % (84/184) of cases (Kappa = 0.48). Influenza vaccinations were underreported in 4 % (7/170) of self-completed questionnaires.

**Conclusion:**

The reliable documentation of vaccinations within the context of the GNC proved to be feasible and thus recommendable at a large scale within the GNC as participants showed high willingness and compliance in providing available vaccination documents. An additional validation by means of documents provided by physicians seems to be possible for more than a quarter of participants. In order to maximize the likelyhood of participants’ of bringing their vaccination certificates it would be sufficient to send a reminder card together with the appointment letter.

**Electronic supplementary material:**

The online version of this article (doi: 10.1007/s00103-014-2050-0) contains supplementary material, which is available to authorized users.

## Abstract (de)

**Hintergrund:**

Daten über den Impfstatus von Teilnehmern in epidemiologischen Studien werden immer dann benötigt, wenn Infektionen oder Immunität als mögliche Exposition oder Outcome betrachtet werden. Wir haben daher die Akzeptanz und Machbarkeit der Impfdatenerhebung durch Erfassung von Impfpässen im Rahmen des Pretest 2 der Nationalen Kohorte untersucht.

**Methoden:**

Die Studie wurde in 3 Studienzentren (Bremen, n = 73; Hamburg, n = 200; Hannover, n = 193) durchgeführt. Um zu testen, ob eine zusätzliche Erinnerung die Bereitstellung der Impfpässe positiv beeinflusst, wurden Personen, die der Teilnahme an Pretest 2 der Nationalen Kohorte zustimmten, einer von drei verschiedenen Erinnerungsgruppen (IG) zugeteilt. Je ein Drittel der Teilnehmer erhielt entweder keine weitere Erinnerung (IG1), eine Erinnerungskarte zusammen mit der Terminbestätigung (IG2) oder eine Erinnerungskarte separat 4 Tage vor dem Untersuchungstermin (IG3). Im Studienzentrum wurden die mitgebrachten Impfpässe unter einem Pseudonym gescannt oder kopiert und in eine Datenbank übertragen. Teilnehmer wurden außerdem gebeten, einen kurzen Fragebogen zur Vollständigkeit der vorgelegten Impfdaten selbstständig auszufüllen. Die Studienteilnehmer in Hannover wurden zusätzlich um das Einverständnis gebeten, Impfdaten über die Hausärzte erheben zu dürfen. Darüber hinaus haben wir die Daten zu Influenzaimpfungen aus den Impfpässen mit den von den Pretest-2-Teilnehmern erinnerten Angaben zu erhaltenen Influenzaimpfungen aus einem Selbstausfüller (ID-Screen) verglichen.

Ergebnisse

Aufgrund unterschiedlicher Startzeitpunkte der Studie wurde das Erinnerungsverfahren nur in den Studienzentren Hamburg und Hannover wie vorgesehen umgesetzt. In Hamburg wurden signifikant mehr Impfpässe in der Erinnerungsgruppe mit einer separat versendeten Erinnerungskarte 4 Tage vor dem Untersuchungstermin (IG3) mitgebracht, verglichen mit IG1 und IG2 (*p* = 0,04). In Hannover wurden dagegen die meisten Impfpässe von denjenigen vorgelegt, die die Erinnerungskarte zusammen mit der Terminbestätigung erhielten. Das Versenden einer Erinnerungskarte hat sich, unabhängig davon, ob sie zusammen mit der Terminbestätigung oder separat übermittelt wurde, positiv, jedoch nicht signifikant, auf das Mitbringen der Impfausweise ausgewirkt: Mit Erinnerungskarte haben 89 % (185/209) der Teilnehmer Impfpässe vorgelegt, ohne Erinnerungskarte 81 % (84/104) der Teilnehmer (*p* = 0,06). Der Datenerhebung über den Hausarzt stimmten 62 % (120/193) aller Pretest-2-Teilnehmer in Hannover zu. Von den 114 angeschriebenen Hausärzten haben 49 Ärzte (43 %) Impfdaten geschickt. 360 Impfpässe mit 5065 Einträgen wurden in eine Impfdatenbank eingegeben. Von diesen sind 4830 (95 %) verwertbar. Die Impfeinträge umfassen den Zeitraum von 1946 bis 2012. Der Vergleich von in den Impfpässen dokumentierten Influenzaimpfungen mit den im Selbstausfüller erinnerten Angaben zeigte eine Übereinstimmung der Daten in 46 % (84/184) der Fälle (Kappa = 0,48). Influenzaimpfungen wurden in 4 % der Fragebögen (7/170) untererfasst.

**Schlussfolgerungen:**

Die hohe Bereitschaft und Zuverlässigkeit der Probanden, ihre Impfpässe vorzulegen, zeigte, dass auf diese Weise eine verlässliche Impfdokumentation im Rahmen der Nationalen Kohorte erfolgen und somit auch in größerem Umfang empfohlen werden kann. Auch erscheint die zusätzliche Validierung über die hausärztliche Dokumentation für über ein Viertel aller Probanden möglich. Um an das Mitbringen der Impfpässe zu erinnern, reicht ein ergänzender Satz in der Terminbestätigung, der standardmäßig eine farbige Erinnerungskarte beigelegt werden sollte.

For research questions in the field of infectious diseases and immunology within epidemiologic studies, data about the vaccination status of the participants are essential. Nevertheless evidence about immunity acquired by vaccinations at the population level is lacking [1]. On account of this insufficient data, research questions like the following have not been investigated so far: (a) vaccination coverage, specifically in adult age [2], (b) vaccine response and immunosenescence [3, 4] and (c) possible interactions between vaccinations, infections and chronic diseases [5, 6]. The German National Cohort (GNC) offers the opportunity to assess vaccination status in combination with the seroprevalences of a large population-based sample throughout Germany and to follow it up over many decades. Other than originally planned, the assessment of vaccinations is not part of the routine data collection of the GNC. Therefore, we conducted an add-on “Level 3” study in Pretest 2 to test the feasibility of vaccination data collection within the GNC but without interfering with the program of the GNC (for details see Ahrens et al. in this issue). Special focus was placed on (1) the acceptance and feasibility of vaccination certificate collection and means of response optimization, (2) the feasibility and benefit of additional vaccination data collection from GPs and (3) the comparison of vaccination data of different sources (vaccination certificates, data from GPs, self-reported influenza vaccinations) regarding completeness and validity.

## Methods

### Recruitment

**Fig. 1:**
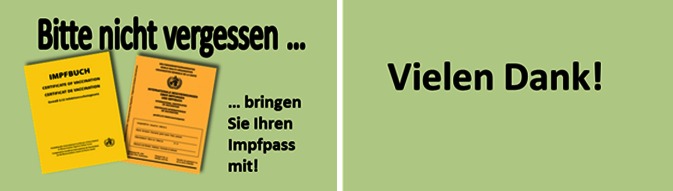
Reminder card

The study was conducted at three sites (Bremen, Hamburg, Hannover). Recruitment followed the standard operating procedure (SOP) of the GNC (Ahrens et al. in this issue). Accordingly, all persons who agreed to participate in Pretest 2 received a standardized invitation letter to confirm the appointment at the study center and to provide further information on how to prepare for the assessment. In this appointment letter participants were also asked to bring their vaccination certificates the day of the examination. To test whether an additional reminder would support participants to think of bringing their vaccination certificates, the use of a colored reminder card was tested (Fig. 1). For this purpose all participants were randomly assigned to one of three different invitation groups (IG). Participants of IG1 received the appointment letter only and no further reminder. Participants of IG2 received a colored reminder card together with the appointment letter, and participants of IG3 were sent this separate, colored reminder card 4 days before the appointment.

### Procedure of vaccination data collection

At the study site, participants gave written informed consent. The vaccination data were scanned electronically (Hamburg, Hannover) or copied (Bremen) under a subject identification number. Personal data like name, address or date of birth were covered before scanning or copying the vaccination certificates. In case participants forgot to bring their vaccination certificate, they were instructed on how to send a copy of the vaccination certificate by mail. In order to assess the feasibility and benefit of additional vaccination data collection from GPs, participants in Hannover were also asked to consent to vaccination data collection from their GPs. If consent was obtained, the practices were contacted by mail. The letter included information about the Pretest 2 of the GNC, participants’ written consent and a documentation sheet for given vaccinations during the last 10 years which could be used in case that data could not be printed out. Finally, all retrieved vaccination data were entered into a database including date of vaccination, name of vaccine, batch number and (combination of) disease(s) to be prevented. If during the data entry questions arose which could not be solved, the entry was to be confirmed as “unfinished” and the reason put into a special field for notes. These unfinished cases were checked and completed as much as possible every other day. New vaccines not yet predefined in the database were added (dynamic list of vaccines). If the date of vaccination (day and/or month) was partly missing, illegible or cut off, the following approach was chosen for coding: the missing day was recorded as 15^th^ of month, the missing month as “June” by keeping the information of recoding in order to be able to differentiate between the real vaccination date and a fictitious date. During the training phase of the personnel (first 50 vaccination certificates) every data entry was checked for quality of data by referring to the copies of vaccination certificates. Afterwards, additional quality checks were carried out for at least 40 % of entered vaccination certificates.

### Questionnaire

A short questionnaire was filled in by participants during their stay at the study center to assess the completeness of the provided data from vaccination certificates. Questions were as follows: if the participant ever possessed a vaccination certificate, if at the time of examination at the study center a vaccination certificate was possessed, the number of vaccination certificates, if other vaccination documents exist, if all received vaccinations are recorded in the presented vaccination certificate and which vaccinations were performed but not documented in the vaccination certificates. One question was related to the attitude towards vaccinations as persons with a critical attitude may be reluctant to provide their vaccination certificate. Every mentioned missing vaccination was coded separately, so if influenza vaccinations of several years were indicated as missing, each vaccination was coded as one missing vaccination. Specific vaccinations mentioned as received during childhood were coded as reported (tetanus as tetanus) otherwise, if not further specified, summarized as “vaccinations received in childhood”.

### Ethics approval

The study was approved by the Ethics Committee of the State Board of Physicians of the German Federal State of Lower Saxony (Ethikkommission der Ärztekammer Niedersachsen) and the federal State of Hamburg.

## Results

### Participation

**Fig. 2:**
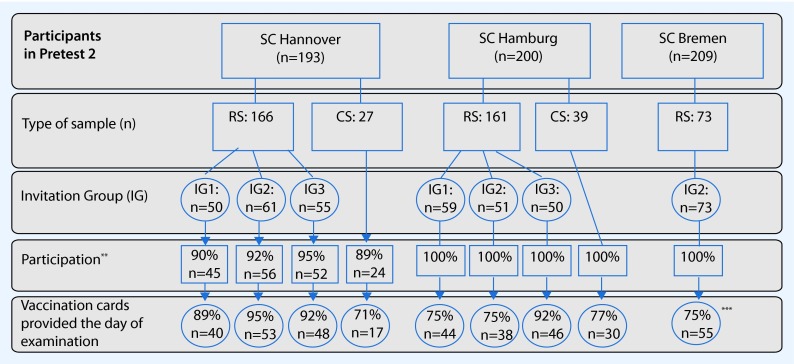
Flow chart of the recruitment and submitted vaccination certificates. *RS =* random sample from local registry, *CS* = convenience sample, consisting of volunteers, e.g. students, friends, colleagues and family members. Invitation group (IG): Appointment letter + … IG1: no further reminder; IG2: a colored reminder card together with the appointment letter; IG3: 4 days before the appointment at the study center a separate, colored reminder card (Fig. 1). *Hamburg: 1 missing case with no information about assigned invitation group, **Participation: Participants were asked for a separate consent for the vaccination data collection in Hannover only, ***Bremen: No documentation sheet about the invitation process available. 75 % of invited persons provided vaccination data the same day of examination or later.

The recruitment process for this feasibility study differed among the participating study centers. On the one hand, the invitation procedure consisting of three different invitation groups was implemented in Hamburg and Hannover only, due to a later starting date in Bremen. Consequently, all participants in Bremen invited to Pretest 2 received by default the reminder card together with the appointment letter. On the other hand, participation proportions for this feasibility study could be calculated for Hannover only, as in Bremen and Hamburg the consent for the vaccination data collection was integrated into the consent form of the Pretest 2 baseline assessment whereas in Hannover participants were asked for a separate consent. Recruitment data and participation proportions per invitation group are shown in Fig. 2. The participation proportions of the random samples are of special interest because the study protocol for the GNC allows random samples only; therefore, participation proportions will be discussed mainly in this respect. Overall, 400 persons were invited to participate in this feasibility study. In Hannover, 92.2 % (153/166) of Pretest 2 participants gave a separate written consent for the vaccination data collection. Again 92.2 % (141/153) of these participants brought vaccination certificates to the appointment. In Hamburg and Bremen, where participants were not explicitly asked for their willingness to participate in this feasibility study, the proportion of provided vaccination certificates was slightly lower: 80.0 % (128/160, one missing) of invited persons in Hamburg and 75.3 % (55/73) in Bremen provided vaccination data.

Considering the overall effect of the reminder card in Hamburg and Hannover, 89 % (185/209) of participants who received a reminder card (regardless if IG2 or IG3) brought their vaccination certificate compared to 81 % (84/104) of those who did not receive any reminder card (IG1; *p* = 0.06). The approach to send a separate reminder card 4 days before the appointment was the most successful; 92 % (94/102) participants of IG3 provided vaccination data compared to 85 % (91/107) in IG2 (*p* = 0.06). Stratifications by study center indicated that in Hannover participants in IG2 provided the most vaccination certificates (95 %), whereas in Hamburg, significantly more vaccination certificates (92 %) were provided in IG3, with no differences between IG1 and IG2 (chi-square^2^: 6.546, df 2, *p* = 0.04).

### Data provided by physicians

Of 193 Pretest 2 participants in Hannover, 120 (62 %) provided written consent for the data collection by general practitioners. In one case the consent was given but the participant had not been in contact with the health care system for a long time so that he could not provide any GP address. In total, 114 practices were contacted of which 43 % (49/114) sent vaccination data. Two practices did not send any data because no vaccination was given during the past 10 years. In most cases the medical staff filled in the paper documentation sheet, with a few providing computerized print-outs.

### Questionnaire

The short questionnaire was completed by 94.7 % (426/450) of participants including the convenience sample. There were considerable differences between the study centers. Whereas in Hamburg all participants completed the questionnaire (200/200), this was the case for 98.9 % (175/177) of the participants in Hannover, and for only 70 % (51/73) in Bremen (data shown in supplement). Thorough data cleaning was necessary because of inconsistent responses. For example, the question, if the participant ever possessed a vaccination certificate was denied by 16 persons of whom 5 persons submitted their vaccination certificate to the study personnel the same day. A problem seemed to exist also if more than one vaccination certificate existed so that participants were not sure to which one to refer. Moreover the high percentage of missing answers for most questions, coded as “don’t know/no answer”, suggests that these questions were difficult to remember and thus not suitable for a self-administered questionnaire.

Of all participants, 96 % had possessed a vaccination certificate once during their life-time, with 91 % possessing one at the moment. There were no differences between random and convenience samples. In all, 37 % of responders indicated that the present vaccination certificate was the first, whereas for 55 % of responders it was the second up to fourth vaccination certificate. In addition, 67 % answered to have brought all available vaccination data to the study center, 54 % included all vaccinations ever received (random sample). Tetanus (18 %), influenza (17 %) and vaccinations received in childhood (13 %) were indicated most often as vaccinations not included in documentation.

The question regarding the attitude towards vaccinations was answered by 416/426 (98 %) participants. The attitude towards vaccinations was assessed by 74 % of responders as positive. Of those 18 % being critical, 45 % indicated fear the side effects of vaccinations and 30 % were concerned that vaccinations might overwhelm the immune system. Among other reasons, responders took the chance to explain more in detail their critical attitude against specific vaccinations like influenza vaccination because of bad experiences. Only one person indicated to be in principle opposed to vaccinations; for 8 % of responders vaccinations were no issues of concern. No statistical difference of attitude towards vaccination could be detected between persons who submitted their vaccination certificate compared to those who did not submit any vaccination data.

### Capturing and availability of vaccination data

Overall 372 vaccination certificates were electronically captured or copied the same day of examination, of which 7 % (27/372) of vaccination certificates were lost primarily because of missing identification numbers (ID) on the copy/scan. The procedure of blackening the personal data before capturing the vaccination data electronically was not followed consequently so that in about 17 % of documents this needed to be worked over subsequently. The quality of vaccination certificates was partly poor, especially the older ones (before 1990) often lacked the names of given vaccines (1700 vaccines), or the date of vaccination. In 5 % (222) of cases, the vaccination date was missing, (partly) illegible or cut off during the process of scanning/copying the vaccination data. Some of these data (121 dates) could be recorded as described above. Further problems encountered were the lack of information about vaccines as single or combined vaccination or the illegibility of documented vaccinations. The median time for data entry was 14.3 min per vaccination certificate (minimum of 0.4 min; maximum of 124.3 min). Including the data of physicians, vaccination data could be obtained for a total of 82 % (367/450) of participants or 79 % (367/466) of all eligible Pretest 2 participants. An overview of submitted vaccination data per study center (number of valid vaccination documents and vaccinations) including the sample characteristics of persons submitting vaccination data is shown in Table 1. The documented vaccinations covered a period from 1946 to 2012.

**Table 1:** Submitted vaccination data and sample characteristics

	Hamburg		Hannover		Bremen		Total	
No. of valid documents	158		155 (162^a^)		47		360 (367^a^)	
No. of vaccinations	2402		1968 (2186^b^)		510		4880 (5098^b^)	
No. of vaccinations/person	15.2		13.9		10.9		14.1	
Mean (95 % CI)	(13.8–16.6)		(12.5–15.3)		(8.7–13.0)		(13.2–15.0)	
**Sample characteristics**	**n**	**%**	**n**	**%**	**n**	**%**	**n**	**%**
Female	90	57.0	87	53.7	25	53.2	202	55.0
Male	68	43.0	75	46.3	22	46.8	165	45.0
20–29 years	23	14.6	18	11.1	3	6.4	44	12.0
30–39 years	15	9.5	17	10.5	-	-	32	8.7
40–49 years	38	24.1	47	29.0	8	17.0	93	25.3
50–59 years	36	22.8	36	22.2	18	38.3	90	24.5
60–69 years	46	29.1	44	27.2	18	38.3	108	29.4
Born abroad	16	10.1	15	9.8	7	14.9	38	10.6
First language German	148	93.7	142	92.8	43	91.5	333	93.0
Married	59	37.3	88	57.5	23	48.9	170	47.5
Single	66	41.8	44	28.8	14	29.8	124	34.6
Full time employed	75	47.5	73	47.7	28	59.6	176	49.2
Monthly household net income (median): 2250 € up to 3000 €	83/146	56.8	73/141	51.8	19/46	41.3	175/333	52.6
**N**	**158**		**162**		**47**		**367**	

*GNC* German National Cohort, *GP* general practitioners, *DEGS* Studie zur Gesundheit Erwachsener in Deutschland, *GEDA* Gesundheit in Deutschland aktuell, *CI* confidence interval

^a^(…): Including data of seven participants in Hannover (4 of random and 3 of convenience sample) who provided physicians’ data only (with 33 documented vaccinations) as no vaccination certificates were available

^b^(…): Including all vaccinations documented by physicians (*n* = 218)

### Comparison of vaccination data provided by participants and physicians

For 40 participants in Hannover vaccination data from vaccination certificates and physicians’ documentation were available with a total of 630 documented vaccinations covering the period from 1956 to 2013. Most frequently documented vaccinations are shown in Table 2. Single vaccinations against poliomyelitis and tetanus were significantly more often, influenza vaccinations significantly less often documented in vaccination certificates than at the physicians’ offices. To assess the quality of vaccination documentation, the data captured by vaccination certificates were compared to data provided by physicians. Using the example of tetanus, 27 persons provided tetanus vaccination information documented in vaccination certificates (125 vaccinations) as well as documented by physicians (54 vaccinations). In all, 30 % (38/125) of tetanus vaccinations documented in the vaccination certificates corresponded exactly with tetanus vaccinations documented by physicians for 20/27 persons (74 %). In these cases the date of vaccination, the kind of vaccine and the producer of the vaccine were documented identically in each document. A total of 6 % (7/125) of documented tetanus vaccinations in the vaccination certificates did not correspond to documented tetanus vaccinations by physicians for 6/27 persons (22 %). Tetanus documentation differed because either the name of the vaccine was missing, not consistent or different producers of vaccines were indicated. In addition, 8/54 (15 %) tetanus vaccinations documented by physicians were not documented in the vaccination certificates which accounts for at least 6 % (8/125) of tetanus vaccinations missing in the vaccination certificates.

**Table 2:** Frequency of vaccinations documented in vaccination certificates compared to vaccinations documented by physicians

Document by	Participant (1956–2012)^a^		Physician (1964–2013)^a^	
Single poliomyelitis	88	20 %	13	7 %
Single tetanus	68	15 %	14	8 %
Tetanus (combined vaccine)	118	27 %	40	22 %
Influenza	33	7 %	66	36 %
Single hepatitis B	22	5 %	8	4 % 21
Hepatitis A + B	20	5 %	14	8 %
No. of vaccinations	446		184	

Base: 40 participants with vaccination certificates and vaccination documentation from physicians

^a^The vaccination certificates covered a period from 1956–2012, the vaccination data sent by physicians from 1964–2013

### Vaccination status

The vaccination coverage of responders (last dose no longer than 10 years ago) for some preventable diseases is shown in table 3.

**Table 3:** Vaccination coverage according to vaccination certificates

		Hamburg		Hannover		Bremen		Total	
		n	%	n	%	n	%	n	% (95 % CI)
Tetanus	Female	74	82	66	76	21	84	161	80 (59–74)
	Male	45	66	49	65	16	73	110	66 (59–74)
	Total	119	75	115	71	37	79	271	74 (69–78)
Diphtheria	Female	69	77	59	68	20	80	148	74 (67–80)
	Male	43	63	46	61	16	73	105	63 (56–71)
	Total	112	71	105	65	36	77	253	69 (64–74)
Polio	Female	50	56	39	45	13	52	102	51 (44–58)
	Male	26	38	30	40	12	55	68	41 (33–49)
	Total	76	48	69	43	25	53	170	46 (41–51)
Hepatitis B	Female	32	36	23	26	3	12	58	29 (23–35)
	Male	17	25	16	21	2	9	35	21 (15–27)
	Total	49	31	39	24	5	11	93	25 (21–30)
Hepatitis A	Female	28	32	23	26	5	20	56	28 (22–34)
	Male	23	33	16	21	1	5	40	24 (18–31)
	Total	51	32	39	24	6	13	96	26 (22–31)
	**N**	**158**		**162**		**47**		**367**	
Measles^a^	Female	19	79	12	63	-	-	31	72 (58–86)
	Male	12	86	11	69	2	67	25	76 (60–91)
	Total	31	82	23	66	2	67	56	74 (64–84)
	**N**	**38**		**35**		**3**		**76**	

Number of persons who received at least one respective vaccination within the last 10 years

^a^Vaccination against measles in Germany recommended since 1974. Base: all participants of the age 20–39 years in the respective study centers who submitted their vaccination certificates

### Comparison of data from a self-completed questionnaire (ID-Screen) versus vaccination data from vaccination certificates

In Pretest 2, the ID-Screen questionnaire, a self-administered questionnaire about infectious diseases, was applied in Hamburg and Hannover (see Castell et al. in this issue). Three questions about influenza vaccination were included: whether an influenza vaccination has ever been received (V1), if yes, in which frequency (V2) and the year of the last influenza vaccination (V3). In total 365 persons answered the influenza questions. Vaccination documents were available for 78 % (284/365) of these participants, which allowed assessing the agreement of self-reported versus documented influenza vaccinations. Half of all responders (184/365) indicated having ever been vaccinated against the flu (Table 4). Agreement with documentations was found for 84 (46 %) of these responders [Kappa 0.48] with up to 20 influenza vaccinations per person. Vaccination was underreported in 4 % (7/170) of cases where the receipt of influenza vaccination was negated but documented in the submitted vaccination records with up to 3 vaccinations per person. The “unvaccinated” responders were mostly vaccinated in 2008–2010 (7/11 vaccinations). The self-reported year of last influenza vaccination was confirmed by vaccination records in 27 % of cases (41/150). In 23 % (35/150) of cases the self-reported year did not correspond to the vaccination records because the year of the last remembered influenza vaccination was before (19 %) or after (5 %) the last influenza vaccination documented in the vaccination records.

**Table 4:** Comparison of influenza data from a self-completed questionnaire (ID- Screen) versus influenza vaccinations documented in vaccination records

	V1: ever vaccinated		V1: confirmed		V2: every year		V3: last year confirmed	
	n	%	n	%	n	%	n	%
Female	103	52.8	53	51.5	29	28.2	26/87	29.9
Male	81	47.6	31	38.3	27	33.3	15/63	23.8
Total	184/365	50.4	84	45.7	56	30.4	41/150	27.3
60–69 years	73/102	71.6	35	47.9	30	41.1	13/63	20.6
Hamburg	95/200	47.5	39	41.1	31	32.6	17/76	22.4
Hannover^a^	89/165	53.9	45	50.6	25	28.1	24/74	32.4

*V1: ever vaccinated* Have you ever been vaccinated against influenza? – yes (ID-Screen questionnaire)

*V1, confirmed* Agreement between reported influenza vaccination (V1) and documented influenza vaccinations in vaccination certificates. *V2* Self-reported regularity of influenza vaccinations in ID- Screen questionnaire (every year, almost every year, less often than every year, only once)*V3, last year confirmed* Agreement between self-reported year of last received influenza vaccination (*n* = 150) and vaccination date found in vaccination certificates

^a^Hannover only: Including vaccination data provided by physicians

## Discussion

Vaccination data collection in Pretest 2 was shown to be feasible as participants were willing to provide vaccination documents. Documentation of vaccination for participants within the GNC would therefore be possible. Considering the completeness and quality of vaccination data it must be said that although the response to this feasibility study was good and much data could be retrieved, there are several limitations to consider. The documented vaccinations covered a period from 1946 to 2012 during which more and more vaccines became available and more vaccination recommendations were given. Not every administered vaccination is documented as vaccination certificates may be forgotten the day of vaccination. Also the fact that vaccinations are administered by different authorities (public health office especially before 1970, different physicians, company physicians, hospitals) accounts for missed vaccination documentation. Some data, like vaccinations given in childhood, are more likely to be missing especially if a person owns already the second or third vaccination certificate. As the comparison of vaccination certificates with data from physicians showed, which could be carried out for about a quarter of participants in Hannover, other received vaccinations may also be missing; however, it is difficult to assess to which extent. Some vaccinations could be found to be more often documented at the physicians’ but not in the vaccination certificate like influenza vaccinations.

Also the comparison of self-reported influenza vaccination (ID-Screen, see Castell et al. in this issue) with the documented influenza data from certificates and physicians was limited by missing vaccination documentation which made it impossible to calculate the extent of overreporting. Underreporting of influenza vaccination occurred in at least 4 % of cases for whom vaccination documents were available. These problems are well-known and discussed in literature. Several studies have shown that neither self-reported vaccinations nor vaccination data based on medical records can be regarded as the gold standard for vaccination documentation [7, 8]. Miles et al. [9] concluded that vaccination cards tend to under-, self-reported vaccinations to overestimate coverage whereas a combination of both overestimated coverage in some studies and underestimated coverage in others ([9], S1565). Rolnick, who described underlying demographic factors for over- and under-reporting, recommends to improve not only vaccination documentation in medical records but also to improve the awareness of patients in order to get appropriate vaccination information ([7], S3933).

As this feasibility study was an add-on within Pretest 2 and was not supposed to interfere with the assessment program of the GNC nor to use much of personnel or other resources there was no possibility for a personal-assisted interview to probe further for missing vaccination data. Despite the data gaps, we found the vaccination coverage of participants to be comparable to figures of other representative long-term studies such as DEGS, where the vaccination data were obtained from vaccination cards and/ or interviews or GEDA, a telephone survey. For example 74 % of Pretest 2 participants had sufficient tetanus vaccination coverage, compared to 71 % in DEGS1 [2] or 72 % in GEDA [10].

The incompleteness of vaccination data extracted from vaccination certificates should be put into perspective to the most important immunological questions addressed within the GNC (introduction), for which at least the vaccinations received during childhood and thus decades ago may be less important. Given the fact that for most vaccinations (except for Hepatitis B) no biomarkers are available to prove that vaccinations were carried out and at the same time the ability of participants to remember all ever received vaccinations is limited and probably also influenced by demographic factors [7], vaccination certificates seem to be at present the best possible population-based data source.

The vaccination data provided by physicians were despite the limitations a useful supplement to the vaccination data of participants. Considering the time and effort required, the data collection from GPs would be feasible and useful only for specific research questions (influenza, or persons without vaccination certificates).

Another aspect of the feasibility of our study refers to the training of the personnel. The electronic capturing and pseudonymization of vaccination data were defined in a standard operating procedure. Nevertheless at least 3 % of all submitted vaccination certificates got lost because of missing identification number. Other problems concerned poor quality of copies, incomplete data due to copying or scanning the data and failure to blacken the personal data dependably. All of these problems can be avoided by further training of study personnel. Also, well-trained staff is needed for the time-consuming data entry of vaccination data into the database.

The use of a reminder card in addition to the standardized appointment letter in which participants were reminded to bring their vaccination certificate to the appointment had a positive effect on the proportion of provided vaccination certificates. If the reminder card should be sent in combination with the appointment letter or separately shortly before the visit in the study center could not be ascertained clearly as results for Hamburg and Hannover were inconsistent. The invitation procedure, especially the delay between appointment arrangements and sending of appointment letters and reminder card was difficult to control and might have varied among the participating study centers. Appointments were mostly made by phone and sometimes short-term, or cancelled and rearranged on short notice which made it often difficult to follow the routine for each invitation group. However, as the reminder card seemed to support participants to think of their vaccination certificates, we suggest sending the appointment letter together with the reminder card, which is not much additional effort.

The short questionnaire which was intended to assess the completeness of provided vaccination data proved to be not suitable as a self-administered questionnaire. As the study protocol for the GNC is already extensive, a staff-assisted interview for these questions will not be feasible. The short questionnaire is therefore dispensable. Nevertheless the question referring to the attitude towards vaccinations was useful, as no statistical difference could be detected between those participants providing vaccination certificates and those who did not, meaning that participants who provided vaccination data were not less critical than persons who did not provide any vaccination data. No bias in this regard could be detected. Reasons why vaccination certificates were not provided may be the lack of documents but also the feeling that providing the vaccination certificate would be too personal.

## Conclusion

We demonstrated the feasibility of collecting vaccination data from vaccination certificates in a population-based study and recommend implementing this approach at a large scale within the German National Cohort in order to allow long-term prospective research on infection and immunity.

## Electronic supplementary material

(PDF 233 kb)

## Abbreviations

GNC: German National Cohort
GP: general practitioners
DEGS: Studie zur Gesundheit Erwachsener in Deutschland
GEDA: Gesundheit in Deutschland aktuell
