# Supplementary material for: Collection of vaccination data in the German National Cohort: Findings of a feasibility study in three study centers
Source: Bundesgesundheitsblatt Gesundheitsforschung Gesundheitsschutz. 2014 Oct 8;57(11):1292–9. doi: 10.1007/s00103-014-2050-0 (PMC4210722; doi:10.1007/s00103-014-2050-0)
Supplement: Supplementary file 1 — (PDF 233 kb) [file 103_2014_2050_MOESM1_ESM.pdf]

Supplement

Table 1: Assessment of vaccination data from vaccination cards submitted in terms of completeness

		Hamburg	Hannover	Bremen	Total
Q1: ever possessed a vaccination certificate?	yes	95.0	96.6	96.1	95.8
	no	3.5	3.4	3.9	3.5
	d.k.	1.5	-	-	0.7
**N**		**200**	**175**	**51**	**426**
Q2: possession of vaccination certificate at present?	yes	88.4	92.9	91.8	90.7
	no	10.5	5.3	8.2	8.1
	d.k.	1.1	1.8	-	1.2
**N**		**190**	**169**	**49**	**408**
Q3: No. of vaccination certificates	1	36.9	43.9	26.7	38.6
	2	36.9	44.6	51.1	41.9
	3	14.9	6.4	8.9	10.5
	4	1.8	1.3	-	1.4
	d.k.	9.5	3.8	13.3	7.6
Q4: Vaccination certificate provided?	yes	88.7	90.4	84.4	88.9
	no	11.3	9.6	15.6	11.1
**N**		**168**	**157**	**45**	**370**
Q5: Other vaccination documents?	yes	19.5	15.4	17.6	17.6
	no	74.0	76.0	78.4	75.4
	d.k.	6.5	8.6	3.9	7.0
Q6: Complete vaccination data brought?	yes	63.5	72.0	62.7	66.9
	no	29.0	19.4	35.3	25.8
	d.k.	7.5	8.6	2.0	7.2
Q7: All vaccinations received included?	yes	52.0	61.7	49.0	55.6
	no	23.0	22.3	33.3	23.9
	d.k.	25.0	16.0	17.7	20.5
**N**		**200**	**175**	**51**	**426**
Q7a: Vaccinations, which are not documented in vaccination certificate (multiple answers possible):					
Tetanus		17.6	22.6	10.0	18.7
Influenza		16.2	17.7	15.0	16.7
Vaccinations in childhood		7.4	11.3	35.0	12.7
Smallpox		10.3	6.5	5.0	8.0
Hepatitis B		2.9	4.8	5.0	4.0
Poliomyelitis		13.2	1.6	5.0	7.3
FSME		2.9	-	-	1.3
Don’t know/no answer		38.2	43.5	40.0	40.7
		**68**	**62**	**20**	**150**
Q8: Attitude towards vaccinations:					
positive		73.3	75.6	71.4	74.0
critical		17.4	16.9	20.4	17.5
opposed against vaccinations		-	-	2.0	0.2
no issue of concern		9.2	7.6	6.1	8.2
**N**		**195**	**172**	**49**	**416**
Q8a: Reason if attitude not positive					
Afraid of side effects (yes)		44.1	53.6	27.3	45.2
Not enough information about necessity of vaccinations (yes)		17.6	14.3	-	13.7
Vaccines may overwhelm the immune system (yes)		29.4	28.6	36.4	30.1
Not enough information about risks of		35.3	21.4	9.1	26.0

vaccinations (yes)				
Other reasons (yes)	32.4	39.3	90.9	43.8
**N**	**34**	**28**	**11**	**73**
